# Ivermectin and the Integrity of Healthcare Evidence During COVID-19

**DOI:** 10.3389/fpubh.2022.788972

**Published:** 2022-03-01

**Authors:** Dónal P. O'Mathúna

**Affiliations:** ^1^College of Nursing, The Ohio State University, Columbus, OH, United States; ^2^Center for Bioethics and Humanities, College of Medicine, The Ohio State University, Columbus, OH, United States; ^3^Cochrane Affiliate, Helene Fuld Health Trust National Institute for Evidence-Based Practice in Nursing and Healthcare, The Ohio State University, Columbus, OH, United States

**Keywords:** healthcare fraud, COVID-19, systematic review and meta-analysis, critical appraisal of literature, ethics, ivermectin, integrity

## Abstract

The COVID-19 pandemic has been characterized by a lack of clear evidence to guide healthcare professionals, the public and policymakers. The resulting uncertainty, coupled with changing guidelines as additional evidence became available, added to the stress and anxiety reported by decision-makers. Research results are key to providing evidence to guide healthcare decisions. Important questions have arisen about whether various interventions are safe and effective. The evidence found guides those making treatment decisions, and influences those selecting interventions for further evaluation in research studies. As the COVID-19 pandemic intensified, the effectiveness and safety of many pharmaceuticals was queried. Ivermectin will be used to explore the ethics of how healthcare evidence must be critically appraised, even, or especially, during a pandemic. This drug is alleged to be effective in treating COVID-19, with various studies and systematic reviews finding supportive evidence. Some of these have now been linked to concerns about fraud or poor research reporting. This article will focus on the scientific literature and how apparently fraudulent studies were published and influenced treatment decisions, on-going research and public health guidelines. Research evidence is critical during emergencies like pandemics, but urgency should not overtake ethical responsibilities to critically appraise (or evaluate) studies as they become available. These responsibilities apply in various ways to editors, peer-reviewers, news media reporters, and those making treatment decisions, including clinicians, policymakers and the general public. While research article authors have the primary ethical responsibility to reject fraudulent or inaccurate claims, the readers of health research must carefully evaluate all publications. To detect and reject fraudulent healthcare claims, readers need critical appraisal skills that match their level of engagement with those articles. The core principles of critical appraisal will be described in the article, and how they can be adapted for different types of readers. Exemplar tools that develop critical appraisal skills will be noted, with reviews of ivermectin's efficacy explored as examples. As stakeholders in healthcare evidence are increasingly able to identify well-conducted and ethical research they will simultaneously be able to spot and reject fraudulent reports and prevent them from influencing healthcare decisions.

## Introduction

The response to COVID-19 has been characterized by uncertainty. This was captured early in the pandemic: “There are no antiviral drugs with proven clinical efficacy, nor are there any vaccines that prevent infection with SARS-CoV-2, and efforts to develop drugs and vaccines are hampered by the limited knowledge of the molecular details of how SARS-CoV-2 infects cells” (1). As the Director of the National Institute of Allergy and Infectious Disease, Anthony Fauci, the Director of the National Institutes of Health, Francis Collins, and others stated in a May 2020 commentary on COVID-19 vaccine research, “We currently know little about what constitutes a protective immune response against COVID-19” (2).

In spite of this lack of information, decisions had to be made by clinicians, patients, and policymakers. Research results play an important role in providing evidence to guide those decisions and are thus important as part of the pandemic response. Without the evidence that research can provide, uncertainty will continue to abound. Without *reliable* and *trustworthy* evidence, people and organizations will make different decisions and give varying recommendations, thereby compounding the confusion and frustration that exists in the midst of uncertainty.

This leads to two ethical imperatives that will be the focus of this article. One is that research should be conducted in the midst of uncertainty. The second is that producers and users of the resulting research findings have an ethical responsibility to carefully evaluate and critically appraise reports of that research. On the first point, the World Health Organization (WHO) in March 2020 published a research roadmap for the pandemic on the basis that “conducting research is linked to ‘a moral obligation to learn as much as possible, as quickly as possible”' (3). In so doing, “research—implemented as policy and practice—can save lives and needs to be integrated into the response from the start” (3).

Much research has been conducted, and this has had many beneficial impacts on preventing and treating SARS-CoV-2 infections. Much more is known now about the virus itself (SARS-CoV-2), the disease it causes (COVID-19), and how infected patients can be cared for at its various stages. At the same time, much remains to be learned. Foremost among the remaining challenges are effective means of preventing infection (prophylaxis) and the identification of effective and safe treatments for COVID-19. Research to develop *de novo* treatments for a novel infection would likely take too long at first, especially given the scale and urgency of the pandemic. Instead, existing interventions already approved for other indications were considered to see if they could be repurposed to treat COVID-19, and experimental medications in development for other conditions were reassessed for potential roles against COVID-19. A variety of candidates were identified for further investigation, primarily those with a track-record of effectiveness against viruses, especially other coronaviruses, or in relieving the symptoms associated with COVID-19. Early candidates included antimicrobial agents like hydroxychloroquine and chloroquine, ivermectin, and lopinavir, approved drugs like interferon and dexamethasone, and experimental agents like remdesivir.

## Time Pressures

Research in any crisis situation has to balance the urgent need for results against the time required to maintain rigorous methods, produce reliable results, and report findings accurately. Attempts to make results available quickly run the risk of allowing data to be published without the necessary checks and balances that are part of how scientific publishing attempts to maintain its standards. At the same time, following standard dissemination practices can cause delays in making potentially life-saving evidence available to practitioners, policymakers and the public who are struggling in the uncertainty. A balance is needed to ensure that making haste does not deteriorate into compromising quality. From a research perspective, “with speed borne of desperation comes risk and confusion—of trials too small to yield answers, of treatments overhyped, and of uncertainty about how to design the best studies possible” (4).

One development that attempts to make research results accessible more quickly is the availability of “preprints.” Manuscripts submitted to journals are typically kept in confidence by authors, journals and reviewers during the peer review process. However, this process can take months or sometimes years. Preprints are manuscripts that are openly available on the internet while the peer review process proceeds. Some journals have their own preprint websites, while other preprint servers are independent of any particular journal. Preprints will typically make it clear that the manuscripts have not been peer reviewed, and therefore clinicians, policymakers, and other readers will need to evaluate the results carefully before accepting or using the findings. While preprints have advantages, their use has increased dramatically during COVID-19 (5), and they have been at the center of a number of controversies about research quality.

This leads to the second ethical responsibility: to carefully evaluate and critically appraise research reports. The importance of this during COVID-19 has been intensified through the unprecedented role of social media and public commentary in communications about what should and should not be used to address COVID-19. The nature of the disease has fueled some of this challenge. Widely varying differences between people in terms of who gets infected after exposure to the virus, the severity of the resulting symptoms, if any, and people's responses to different treatment protocols have led to a dizzying array of anecdotal reports about people's experiences with COVID-19. Social media now gives almost anyone the means of promoting or criticizing any recommendation for COVID-19. Such reports can put pressure on practitioners, policymakers and politicians to promote various treatments, with or without reliable evidence to support the claims. In such an atmosphere where anything can be promoted, misinformation and fraud can flourish. Tragically, this has happened, worsening the uncertainty and confusion in the midst of this devastating pandemic. Hence, the need to carefully evaluate all recommendations about COVID-19.

The importance of these issues will be demonstrated here in relation to one of the drugs widely promoted both to prevent and treat COVID-19: ivermectin. Some of this discussion overlaps with hydroxychloroquine, another drug that received much attention after the Presidents of the US and France made statements that were taken to support their effectiveness (6). Ivermectin has been used so much that when formulations made for humans became scarce, people started using veterinary formulations. This led the US Food and Drug Administration (FDA) to tweet, “You are not a horse. You are not a cow. Seriously, y'all. Stop it” (7). The tone of this tweet contrasts dramatically with the more scientific language typically used by such agencies to draw attention to concerns about a medication, yet is an indication of how serious the situation has become (8).

## Ivermectin

Ivermectin is chemically derived from an active ingredient isolated from a bacterium found in Japan (9). It came to medical prominence when it was discovered to be an effective treatment for onchocerciasis (or river blindness), a disease transmitted by a parasitic worm (10). This plagues millions of people living in low-income tropical regions around the world, although ivermectin has led to its elimination in some countries. A well-documented partnership with Merck & Co. Inc. led to its free distribution in endemic regions for as long as necessary, which has been hailed as an exemplary humanitarian success story and contributed to its inventors sharing the 2015 Nobel Prize in Physiology or Medicine (10). It is FDA-approved for oral use in treating parasitic worms and topically for head lice and other parasites. Billions of doses have been distributed for use in animals and humans, with an excellent safety record (11). It has since been shown to be a safe and effective treatment for several parasitic diseases in humans and animals.

Ivermectin continues to be investigated as a potential treatment for additional infectious agents, which led to it being tested against SARS-CoV-2. In April 2020, a peer-reviewed article was published showing that ivermectin prevented the replication of SARS-CoV-2 (12). The authors highlighted that they had conducted an *in vitro* study in the laboratory, not one in animals or humans. In addition, the doses used were 50–100 times higher than the equivalent doses approved for use in humans. In spite of this, public interest was fueled by media reports which sometimes did not describe both of these crucial details (13).

Interest in ivermectin for treating COVID-19 was particularly high in Latin America where it is used widely against parasites. This resulted in shortages for its approved uses, which points to another ethical issue with the inappropriate use of a medication without evidence of benefit (11). The resulting scarcity of ivermectin led to people in Latin America using veterinary ivermectin. This became so widespread that the FDA issued a warning about the differences between human and animal products (14). Over a year later, poison control centers in the US saw a dramatic increase in people overdosing from using veterinary ivermectin to treat COVID-19 (15). That was when the FDA released the tweet quoted earlier.

Ivermectin has impacted not only individuals, but also whole populations through public policy for COVID-19. Policymakers in Latin America faced ethical dilemmas over how to address their situation. Victor Zamora, Peru's Health Minister, stated in May 2020 that while they were developing guidelines and policies on COVID-19 for clinicians, they did not have time “to wait for scientific evidence” (16). As a result, Peru added ivermectin to its COVID-19 clinical guidelines. This was followed by Bolivia, with its health minister acknowledging that ivermectin “does not have scientific validation in the treatment of the coronavirus” (17). These recommendations were followed in 2020 by similar ones in Brazil, Chile and other Latin American countries (17). The subsequent explosion of COVID-19 cases in Latin America points to the consequences of getting this wrong (18). In spite of widespread distribution of ivermectin, both by prescription and through black market sources, COVID-19 case numbers and deaths climbed during 2020, with ivermectin eventually being removed from Peru's clinical guidelines on October 12, 2020 (16). Many factors contributed to this, among which Brazilian researchers have included widespread misuse of ivermectin and official communications which were not based on scientific evidence (18). In spite of this, doctors reported being pressured to prescribe ivermectin, while researchers reported difficulties conducting rigorous randomized controlled trials (RCTs) because patients did not want to take the chance of being assigned to a group that didn't receive ivermectin. One Peruvian researcher reflected that, “I think people have lost faith in science … and it has been very, very bad for us in Latin America” (cited in 16).

## Problematic Publications and the Need for Critical Appraisal

The growth in popularity of ivermectin during COVID-19 can be linked to some very problematic publications. Fraud is particularly difficult to demonstrate because it implies a deliberate intention to deceive and to personally gain from such deception. For that reason, this article will refrain from using the term fraud except when it has been demonstrated through thorough investigations. Instead, terms will be used such as poor quality, methodological problems, or high risk of bias. These can be demonstrated regardless of the intentions of the authors or researchers.

The problems with ivermectin publications began early in the pandemic when an April 2020 preprint reported findings from a large medical database owned by a US company called Surgisphere (19). The publication's findings were based on information contained in a database which reportedly collected details from the electronic health records of COVID-19 patients in hospitals around the world (20). Surgisphere's owner, Sapan S. Desai, co-authored this preprint which reported that COVID-19 patients who received ivermectin had a death rate of 0.7% while it was 18.6% for those not receiving ivermectin (19). The authors posted a new version of their study shortly afterwards in which they compared matched patients with one another, rather than reporting overall averages (21). In this analysis, the death rate was 1.4% in patients receiving ivermectin compared to 8.5% for matched patients who did not receive ivermectin.

As ivermectin was becoming widely used for COVID-19 in Latin America, Carlos Chaccour, a Venezuelan physician and researcher who had prescribed and researched ivermectin in South America and Africa, identified serious discrepancies in the Surgisphere data (16, 22). He noted that the data included 52 COVID-19 patients receiving ivermectin before it was recommended for COVID-19. Three patients on ventilators were included from African hospitals at a time when only two COVID-19 patients had been identified in Africa, and neither had received ventilator support. A third patient was later identified, and did not require a ventilator. Chaccour's experience in Africa also led him to question whether many African hospitals had the electronic patient record systems that Surgisphere claimed they used to collect data.

In May 2020, Surgisphere data formed the basis of two other peer-reviewed articles in two of the leading medical journals in the world: *The New England Journal of Medicine* (23) and *The Lancet* (24). The first of these articles concluded, based on data from almost 9,000 patients, that COVID-19 patients with heart disease had worse outcomes, a finding that corroborated many case reports with small number of patients (25). Later that month, the same research group's *Lancet* article was based on data from almost 100,000 patients in 1,200 hospitals around the world. It reported that the death rates and incidence of heart problems increased in COVID-19 patients taking hydroxychloroquine, another highly popular but controversial medication for COVID-19. The next day, WHO halted the use of hydroxychloroquine in a large international clinical trial it was sponsoring, with other research trials stopping also (22).

Similar to the ivermectin preprint, some careful readers noticed that the two Surgisphere publications contained data from more Australian COVID-19 patients than had been diagnosed at that time, which led to *Lancet* publishing a correction (26). Investigative journalists noted that prior to 2019 Surgisphere was a medical textbook publishing house and raised questions about how it could transition so rapidly into a data analytics company with such a massive database (27). They contacted Australian hospitals about how they contributed patient data, but none had any knowledge of Surgisphere. Additionally, journalists noted that data on race was reported for countries where such data is not collected. Surgisphere refused to allow independent validation of the data, claiming this would violate confidentiality and client agreements (20). These and other concerns led to the two published articles being retracted by all of the co-authors except Sapan Desai, the owner of Surgisphere (20). The co-authors admitted they had not viewed or analyzed the Surgisphere data and were unable to verify the articles' analyses. They thus admitted to having violated basic publication ethics, exemplified by an article published almost 10 years earlier: “It is incumbent upon the publisher, editors, authors, and readers to ensure that the highest standards of scientific scholarship are upheld” (28). Ironically, the first author of this article was Surgisphere's owner.

The impact of these retracted publications has been immense. Ian Kerridge, an Australian bioethicist, noted that that the “whole event is catastrophic,” causing problems in many areas, including for the journals involved, the integrity of science, and the notion of evidence generation (cited in 20). The hydroxychloroquine trials were restarted after the retractions, and their results have contributed to the evidence showing that this is not an effective COVID-19 treatment (29). The multiple inconsistencies and problems in these articles have led to all the Surgisphere COVID-19 publications being viewed as potentially fraudulent (27). The Surgisphere ivermectin preprint was removed from its website by some of the co-authors who stated that it was not ready for peer review (17). The whole episode highlights the problems when healthcare policy is not based on reliable evidence, and when peer review is not thorough enough to identify problems in evidence reports. It also points to the crucial importance and value of readers carefully evaluating research reports before accepting their findings.

## Critical Appraisal

Concerns about the quality of research and other evidence, especially in healthcare, can be addressed with critical appraisal tools and reporting guidelines (30). Each approaches the issue from a different perspective, but together have led to an extensive collection of tools and guidelines that can help anyone evaluate the strengths and weaknesses of a piece of evidence. Critical appraisal is the process of carefully and systematically evaluating the quality of a piece of research to determine its value, validity and relevance. Critical appraisal skills are essential for anyone using healthcare literature, whether professional practitioners, policymakers, media reporters, or patients. Reporting guidelines are provided to guide authors of research reports to include all the elements essential for readers to be able to critically appraise those reports. Since these guidelines list all of the essential elements of a high-quality research report they can also help during critical appraisal. Specific guidelines and appraisal tools are available for every type of research study (RCT, systematic review, survey, qualitative study, etc.) and some are specific to distinct research topics. Many of these tools are freely available on the internet (31). One tool is specifically designed to detect fraud (32).

Critical appraisal tools and reporting guidelines have developed, in part, as a response to concerns about the quality of healthcare research publications. Some journals now require authors to document how their manuscript adheres to the reporting guidelines that apply to their research methodology when they submit a manuscript for publication. The manuscript will then be sent for peer review, where independent researchers have an opportunity to evaluate and critically appraise a study and advise the journal editors on whether the article should be published and if revisions are required before that. Peer review has limitations, as it depends on suitably qualified researchers volunteering their time to conduct a careful evaluation (33). The process takes time to conduct and allow authors to respond to reviewers' feedback. The cycle may be repeated, and in the end the journal may decline to publish the manuscript, and then the authors have to start with another journal. During the COVID-19 pandemic, changes were introduced to speed up the peer review process, with some concerns about whether or not this compromised the integrity of the process (34). This is particularly so with preprints, as already discussed.

## Systematic Reviews and Ivermectin

Evaluating treatments for a new disease is particularly challenging because the situation begins with a lack of information, but as research takes off, the challenge switches to one of keeping up with the accumulation of new information. With something as widespread as a global pandemic, the challenge is intensified. Over the last few decades, the health and medical literature has seen a literal explosion in the numbers of articles being published (35). Research related to COVID-19 led to a further unprecedented increase in publications (5). Any attempt to summarize such literature faces the additional problem that new information can become available before an article is published. For example, a Cochrane systematic review of RCTs using ivermectin to treat COVID-19 was published in July 2021. It included 14 RCTs, but noted that 31 trials were ongoing at that time, and the reviewers were waiting for information from an additional 18 trials which might meet inclusion criteria (36). This is a rapidly growing field of research, with new evidence becoming available on a regular basis (37).

However, systematic reviews and meta-analyses play a vital role in summarizing such information. When several studies are published on a similar topic, they can be summarized in a systematic review. If the studies are similar enough and contain quantitative data, those can be combined statistically in a meta-analysis. Cochrane systematic reviews are recognized internationally as being of high quality both in general (38) and in specific fields, like oncology (39). Variation in the quality of non-Cochrane systematic reviews points to the need to critically appraise all systematic reviews before their evidence is used to inform practice or policy (39).

Given the interest in ivermectin for COVID-19, and to evaluate the quality of ivermectin systematic reviews, PubMed was searched on 31 August 2021 using the terms “ivermectin,” “COVID-19,” “systematic review,” and “meta-analysis.” This identified 19 publications, of which 11 were excluded (4 were reviews of multiple interventions, 3 were letters or commentaries, 2 were clinical guidelines, and 2 were systematic reviews of non-human trials). Of the 12 relevant systematic reviews, 4 included multiple interventions or were network meta-analyses and were not examined further here. Two additional systematic reviews were identified from the reference lists of the articles identified, giving a total of 10 systematic reviews and meta-analyses of ivermectin for the prevention or treatment of COVID-19. A complete list of the 21 references is provided in Supplementary Material A.

Some variability among the 10 systematic reviews and meta-analyses addressing ivermectin's effectiveness with COVID-19 is to be expected. One completed its search on 31 August 2020 and included 4 non-randomized controlled trials (40), while another searched on 26 May 2021 and included 14 RCTs (36). The 10 systematic reviews and meta-analyses included also varied in the outcomes they addressed, with one examining only ivermectin's prophylactic use (41), two examining only mortality (42, 43), and most examining several outcomes. Such variability is completely appropriate as reviewers will have different questions leading them to review the literature. The existence of a relatively large number of systematic reviews on the same topic points to the need for more coordination between reviewers, especially given the variability in the methods chosen. A systematic review of these reviews, called an overview, is warranted, but will be challenging given this variability between reviews and how rapidly new ones are appearing.

This article will focus on aspects of variability that point to problems in the quality of some systematic reviews and meta-analyses. This supports the need for readers to critically appraise the results and conclusions of systematic reviews and meta-analyses before applying them to practice or policy. This article will also point to the way potentially fraudulent trials can have an enduring impact on healthcare policy and practice when included in systematic reviews, further highlighting the need for critical appraisal and evaluation of risk of bias when conducting systematic reviews. The concern here is how relatively similar systematic reviews can come to diametrically opposing conclusions. For example, Kory et al. published a review and meta-analyses (search conducted 12 December 2020) with its abstract concluding:

“*Meta-analyses based on 18 randomized controlled treatment trials of ivermectin in COVID-19 have found large, statistically significant reductions in mortality, time to clinical recovery, and time to viral clearance. Furthermore, results from numerous controlled prophylaxis trials report significantly reduced risks of contracting COVID-19 with the regular use of ivermectin. Finally, the many examples of ivermectin distribution campaigns leading to rapid population-wide decreases in morbidity and mortality indicate that an oral agent effective in all phases of COVID-19 has been identified”* *(**44**)*.

Another systematic review (search conducted 25 April 2021) conducted a meta-analysis of 15 RCTs measuring mortality and concluded that ivermectin compared to no ivermectin reduced the risk of death by an average of 62% (37) with the GRADE approach used to evaluate this as “moderate-certainty” evidence. Another systematic review (search conducted 22 March 2021) of 10 RCTs concluded “that ivermectin did not reduce all-cause mortality” (45). The Cochrane review (search conducted 26 May 2021) included 14 RCTs and concluded that it was uncertain whether ivermectin increased or decreased mortality and the evidence was “very low-certainty” (36). The authors noted that the completed studies were small and “few are considered high quality.” They concluded that “reliable evidence does not support the use of ivermectin for treatment or prevention of COVID-19” except within well-designed RCTs (36).

## Comparing Systematic Reviews

We will examine in detail the review and meta-analyses with the most favorable findings (referring to it as the Kory review, 44) and compare it to the Cochrane review (36). As noted earlier, various critical appraisal tools and reporting guidelines are available to guide authors and readers. The Preferred Reporting Items for Systematic Reviews and Meta-Analyses (PRISMA) 2020 guidelines is a 27-item checklist for reporting systematic reviews (46). The Kory review satisfied 8 of those criteria fully, and 2 partially. For example, they gave no descriptions of their search strategy, their inclusion and exclusion criteria, the methods of their meta-analyses, etc. A similar 12-item checklist exists for systematic review abstracts (47), with the Kory review satisfying one criterion, and partially meeting a second. Completed PRISMA checklists for the Kory review are provided in Supplementary Materials B, C. This contrasts with the Cochrane review which included almost all elements of both PRISMA checklists. The Cochrane abstract was missing two items (its funding source and protocol reference) and some further details sought in the PRISMA for Abstracts checklist (details in Supplementary Materials D, E). The Cochrane review's full text provided all this information and therefore these omissions are inconsequential. The Cochrane review is of the highest quality, while the Kory review fails to provide much of the information important to its critical appraisal.

Critical appraisal tools focus on the validity, impact and applicability of systematic reviews, and come in various formats, like A MeaSurement Tool to Assess systematic Reviews (AMSTAR 2) checklist (48), and the Critical Appraisal Skills Programme (CASP) checklists (49). The latter also include more open-ended questions that stimulate reflection on the details of a review's methods. For example, question 5 of CASP's systematic review checklist asks if it was reasonable for the review to combine the results of included studies. On the surface, it appears that this was the case in the Kory review. However, a detailed examination leads to the opposite conclusion. A reader can quickly see if a review or meta-analysis satisfies the PRISMA 2020 guidelines, and if not, a more careful examination of its details is warranted. This can involve examining the original studies included in the review. If inconsistencies and methodological problems become apparent, the review's recommendations should be questioned, if not dismissed.

The Kory review divided its examination of ivermectin clinical trials into three areas: (i) prophylaxis trials to prevent COVID-19, (ii) clinical trials with mildly ill COVID-19 outpatients, and (iii) trials in hospitalized patients. Each will be examined to highlight methodological and reporting problems. In the interests of space, only the RCTs included in the Kory review will be discussed as RCTs generally provide the highest level of clinical evidence in evaluating the efficacy of healthcare interventions (50).

### Prophylaxis Trials

The Kory review included three prophylaxis RCTs and five observational trials. The first RCT was a preprint which was subsequently withdrawn for reasons discussed in detail below (51). In its prophylaxis trial, 200 people with regular contact with COVID-19 patients (either as healthcare workers or household members) were randomly assigned either to receive ivermectin and wear PPE, or to a control group wearing PPE only. Significantly more people in the control group became infected as tested by PCR (10 vs. 2%, *p* < 0.05). At the time of the Kory review, this trial was appropriate to include.

The second trial was peer-reviewed and randomized 340 household contacts of people with COVID-19 to receive either ivermectin on days 1 and 3 or no intervention (52). The study was at high risk of bias because it was unblinded (open label), and the report provided insufficient information on randomization, allocation concealment, missing outcome data, and how the outcome was measured. PCR testing was limited and performed on only 16 participants, with all others evaluated by their clinical symptoms. Given these limitations, the study found significantly fewer people infected in the ivermectin group (7.4 vs. 58.4%, *p* < 0.001).

The third RCT was a preprint and described in the Kory review as following the “Carvallo IVERCAR protocol” and finding that 12 mg ivermectin protected healthcare workers significantly (53). However, the Kory review initially described this protocol as containing “the medicines” ivermectin and carrageenan. This makes this trial ineligible for a review of ivermectin alone since the effects of ivermectin cannot be separated from those of the other medicine. Yet four times in the text and three times in their Table 3, the Kory review described the intervention only in terms of ivermectin. The RCT (53) and original Carvallo observational study (54) described the intervention as a combination of ivermectin and iota-carrageenan, the latter selected because of its antiviral activity and potential synergistic activity. Neither of these trials studied ivermectin alone and should not have been described as if they did and therefore should not have been included in this review. In the same paragraph, the Kory review stated that the Carvallo team conducted a much larger follow-up observational trial that found remarkable protection from ivermectin. This observational trial used a completely different intervention containing ivermectin, enoxaparin (an anticoagulant), aspirin, and dexamethasone (55). This trial likewise should not have been included here, and it was totally inappropriate to describe its effects as being due to ivermectin, especially given that dexamethasone is an effective COVID-19 treatment (56).

Given this, only one RCT fits the Kory review's own criteria (now that one has been withdrawn). This was the conclusion reached in the Cochrane review (36). They included one RCT (52), but because of its high risk of bias, did not include it in their primary analysis or summary of findings tables. While it reported evidence of benefit, it also found evidence of harm through more adverse events in the ivermectin group (not reported by the Kory review).

### Outpatient Trials

The second section in the Kory review examined trials with mildly ill outpatients and included seven RCTs and four case series. However, of these seven RCTs, only two met the Kory review's own inclusion criteria for this section. Three of the trials used ivermectin plus doxycycline, a tetracycline antibiotic which makes them ineligible for an ivermectin-alone review (57–59). One trial clearly included hospitalized patients, including those with breathlessness and/or hypoxia (60), while another described the participants as patients but did not specify if they were outpatients or inpatients (61). That trial gave the control group Lopinavir/Ritonavir which is used to treat COVID-19 and complicates the analysis. Only two RCTs clearly matched this section's inclusion criteria (62, 63). The Cochrane review included both these trials in its analysis of outpatients.

### Trials With Hospitalized Patients

According to the Kory review, hospitalized patients participated in six RCTs, five observational studies and one database analysis. The largest of the RCTs was the subsequently withdrawn study to be discussed below, which the Kory review analyzed as two separate trials (51). Three of the other RCTs used ivermectin plus doxycycline (58, 64), so should not have been included. Another RCT used appropriate methods, but 28.9% of the participants had negative PCR tests for COVID-19 and thus were not eligible for inclusion (65). One RCT (66) enrolled outpatients with mild symptoms and therefore should have been included in section (ii), as it was in the Cochrane review. Thus, only one of the Kory review's RCTs (apart from the withdrawn one) satisfied their own inclusion criteria for this section (67).

The inaccuracies continued into the meta-analyses conducted in the Kory review. For example, their meta-analysis for mortality listed four observational studies and six RCTs. These six included the withdrawn study, two that used ivermectin plus doxycycline (57, 58), the one where almost one-third of the participants had negative PCR tests for COVID-19 (65) and a fifth which clearly described itself as an observational study involving comparisons with historic case controls (68). Thus, only one of these studies was truly eligible for this meta-analysis (60). The Kory review reported summary data for a meta-analysis of the observational studies and a second for the RCTs, and then combined both of these groups for an overall summary. The *Cochrane Handbook* is emphatic (the bolded text is theirs) that RCTs and non-randomized studies “**should not be combined in a meta-analysis**” (69). Meta-analysis of observational studies can be done, but is challenging for many reasons, and requires careful analysis of the risk of bias and heterogeneity among the studies, something not done in the Kory review (50).

In summary, the Kory review's findings that were favorable toward ivermectin can be traced to at least five flaws in how they conducted their review. A rapid comparison of their review to the PRISMA 2020 guidelines should have alerted peer-reviewers, publishers and readers to serious problems in their report which should not have been published until these were corrected. These flaws include: (A) They included studies that used inventions where ivermectin was given in addition to other drugs (some known to be effective against COVID-19), thereby adding bias toward effectiveness. (B) A trial with outpatients was included among their group of trials with hospitalized patients, and a trial with hospitalized patients was included in their outpatient group. (C) They included trials with relatively large numbers of patients who did not have COVID-19. (D) A large RCT with positive effects for ivermectin was later withdrawn. While the Kory review team could not have anticipated this, their response introduced further problems to be discussed in the next section, along with the fifth flaw (E).

The Cochrane review included the three outpatient trials noted above, plus another published after the Kory review's search (70). This was the largest RCT to date and found no significant benefits from ivermectin. This contributed to the Cochrane review's conclusion that we do not know if ivermectin is effective with outpatients based on the studies providing only low or very-low uncertainty evidence (36). For hospitalized patients, the Cochrane review found nine studies, also evaluated as low or very-low uncertainty evidence, leading to their conclusion that “Ivermectin compared to placebo or usual care may make little or no difference to improving patients' condition 28 days after treatment” or to how long they remain hospitalized (36). Critical appraisal of the Cochrane review shows that its conclusions can be relied upon.

## The Withdrawn Ivermectin Study

One final area needs to be addressed. As noted, the Kory review (and many others) included one of the largest RCTs of ivermectin at the time. It produced remarkable results, reducing COVID-19 death rates by more than 90%. It provided the Kory review the largest number of hospitalized COVID-19 patients, a sizeable number for the prophylactic section, and impacted all the meta-analyses through its data favoring ivermectin. The Kory reviewers could not have known that it would subsequently be withdrawn, initially “due to ethical concerns,” but later changed to “an expression of concern” that is “now under formal investigation.” The Kory review team have since published a letter where they reanalyzed their data without the withdrawn study and stated that their results were largely unaffected (71). However, they added new data from three additional studies into their meta-analyses without declaring this or providing references for the new studies. They also removed another study (68) from their meta-analysis, which was appropriate, but not declared in their letter. The additional studies they included reveal further problems in their work.

The reanalysis included one observational study and two RCTs. We will examine just the RCTs. One was identified as “Seet,” adding data to the prophylaxis meta-analysis. PubMed identified one RCT of ivermectin by Seet et al. with the same data (72). It randomized 3,037 asymptomatic participants to five groups: ivermectin, hydroxychloroquine, povidone-iodine spray, zinc plus vitamin C, or vitamin C alone as control. The primary outcome was laboratory-confirmed SARS-CoV-2 infection, with the ivermectin group having 398 infections in 617 participants compared to 433 out of 619 participants in the control. This difference was not statistically significant. However, the Kory reanalysis did not use this data (71). They used a secondary outcome, “symptomatic COVID-19,” which had statistically significant differences: 32 in the ivermectin group compared to 64 in the control (*p* = 0.0034). This was inappropriate as the Kory meta-analysis examined numbers of infections, not numbers of symptomatic patients.

The second RCT included in the reanalysis was identified as “Rezai.” PubMed revealed one RCT of ivermectin with the last author's name Rezai, and similar data (73). The reanalysis included this study in the meta-analysis of time to recovery, but reported 53 people in each group. The study actually included 35 in the ivermectin group and 34 in the control group. This was accurately reported in the mortality meta-analysis. However, this study should not have been included at all. In this RCT, 69 participants received standard of care which included hydroxychloroquine and/or lopinavir/ritonavir, but without details as to who received which. The intervention group also received ivermectin. COVID-19 PCR tests were performed on 25 participants, of which 9 were negative. Thus, over one-third of the participants may not have had COVID-19. The study's inclusion criteria included patients with symptoms *compatible* with COVID-19, which makes it ineligible as a study of *confirmed* COVID-19 patients. This, and the use of co-interventions in unclear ways, means this study should have been excluded, as it was from the Cochrane review (36). Their reanalysis thus contributes a fifth flaw to those identified above: (E) they selected the wrong outcome from one study which led to the inclusion of data that was more favorable toward ivermectin, but further invalidated their findings.

Returning to the large RCT of ivermectin that was subsequently withdrawn, it was first made available in November 2020 as a preprint and subsequently updated twice (51). Its large number of participants had a significant impact on all systematic reviews including it. A master's student at the University of London was studying it and decided to conduct an in-depth critical appraisal. This revealed serious concerns, including large sections of its introduction being apparently copied from other sources (74). The student contacted researchers specializing in fraud in scientific publications and requested their involvement (75). The preprint included a link to the study's raw data allowing its details to be examined by others (76). These researchers identified many discrepancies between the data and the preprint (77). For example, the preprint stated that subjects were all over 18 years old, but the raw data included patients under 18; the raw data included some collected before the study was reported to have begun; and most of the descriptive data (like mean ages, gender, etc.) differed between the raw data and the preprint. More detailed scrutiny revealed that dozens of data cells appeared to have been copied from one participant to another, with small changes introduced in various places. The author of another systematic review (37) that included the preprint's data stated that they had corresponded with the preprint authors to clarify some data and had no reason to doubt the study's integrity (75). The lead author of the study continues to defend his study and that the apparent problems can be explained (75).

The withdrawal of this large study means that uncertainty continues over ivermectin's effectiveness in COVID-19. What remains are several small studies, many of which did not find ivermectin effective for many outcomes. At the time of writing, the evidence from rigorous and ethical research does not support the use of ivermectin in preventing or treating COVID-19. The Cochrane review and WHO have reached the same conclusion, adding that ivermectin should be studied further in RCTs (78). Other professional organizations in Europe, North America and India support this conclusion (36). Even Merck, which manufactures ivermectin, has concluded that there is “no meaningful evidence” to support using its product against COVID-19 (79). Many trials are under way, and will hopefully provide more definitive answers on ivermectin and other treatments for COVID-19 (80).

## Conclusion

Research remains crucial to healthcare, including during a pandemic involving a new disease. The urgency for effective interventions when none exist calls for careful, rigorous research that should be reported as rapidly as possible. Innovation here can be beneficial, such as developments in living systematic reviews that are updated as soon as new evidence becomes available (81). Network meta-analysis is another innovation where several interventions used to treat the same condition can be compared by including trials that directly compared different pairs of treatments and controls and, when appropriate, comparing interventions when head-to-head comparisons were not conducted (82). However, these innovations introduce additional complexity that requires specialized expertise to analyze and apply the findings accurately to practice and policy.

At the same time, the urgent need for evidence should not be allowed to recommend, provide or support the use of treatments without rigorous evidence that has been thoroughly critically appraised. Original research should be critically appraised by all those using it, whether in healthcare practice, in policymaking, or in the media. Many tools and guidelines are available to allow rapid critical appraisal (for example, 49), and if concerns are identified, a more thorough examination undertaken. This should happen during a systematic review, but those reports must also be critically appraised. The need for all users of healthcare evidence to conduct critical appraisal is clear. However, in-depth analysis such as that carried out here and by others (74, 77) takes time that busy clinicians and policymakers may not have. This sort of detailed appraisal has an important place, but rapid appraisals can be carried out using checklists like PRISMA 2020 and the many others for different study methodologies (31). Users of healthcare literature should use relevant checklists while reading articles until they become completely familiar with recommended guidelines. This will both facilitate their development of critical appraisal skills and allow readers to decide if an article's findings and conclusions are reliable, valid and trustworthy. Those articles that fail to adhere to these standards should be disregarded, or used cautiously, and only articles that do comply with applicable standards relied on for clinical or policy decision-making.

Critical appraisal skills also need to be taught during professional training so that students learn early on that they cannot rely on a study just because it has been published. When teaching research skills, articles that report research well and accurately should be compared with those that do not. Continuing education should also provide opportunities for developing and updating critical appraisal skills. In addition, the peer-review process needs to be strengthened. The importance of this process to the integrity of healthcare literature has to be reiterated. Practical steps have been developed to guide peer reviewers (83). However, the process is volunteer-based and highly dependent on the time and effort reviewers put into their peer review. At a minimum, reviewers should see that their role includes critical appraisal of the study, and use the reporting guidelines available for the type of study they are peer reviewing. They must also be committed to address concerns or suspicions they may have, even if that leads to further investment of time into the review. Publishers and professional societies should also consider ways to further incentivize and support the peer-review process given how crucial it is to supporting the integrity of their publications and professions.

These suggestions only scratch the surface of how critical appraisal needs to be promoted. Busy clinicians, reporters, patients and policymakers need support to identify which reports can be trusted and which not. The current public debate over COVID-19 recommendations and interventions has highlighted the importance of explaining how evidence must be and can be appraised. This has reached the point where in July 2020 a former editor of *BMJ*, a leading medical journal, asked whether it is “Time to assume that health research is fraudulent until proven otherwise?” (33). Some empirical evidence suggests that the answer may be yes (84). When the world is scrambling for something to treat a pandemic, the pressure to recommend anything can be intense. The author of one systematic review (85) told *Nature* that many of the ivermectin trials his team scanned were “likely to be flawed or statistically biased” (75). Whether the problem is time pressure, sloppiness, incompetence or fraud, it requires a careful and thorough evaluation of all research reports for their methodological rigor and ethical quality before being used to influence policy or practice (86).

While it is great that graduate students, investigative journalists, and internet evidence sleuths have identified problems in healthcare studies, the integrity of healthcare literature should not be left to happenstance examinations. The reputations of healthcare professionals, researchers and health policymakers are at stake, and so too is trust in the evidence generation and scientific dissemination process. Evidence and ethics must be combined to ensure that the needs and safety of patients are given the highest priority in the production and application of healthcare literature. Part of this involves teaching all users of healthcare evidence to critically appraise everything they read about their health.

## Author Contributions

The author confirms being the sole contributor of this work and has approved it for publication.

## Conflict of Interest

The author declares that the research was conducted in the absence of any commercial or financial relationships that could be construed as a potential conflict of interest.

## Publisher's Note

All claims expressed in this article are solely those of the authors and do not necessarily represent those of their affiliated organizations, or those of the publisher, the editors and the reviewers. Any product that may be evaluated in this article, or claim that may be made by its manufacturer, is not guaranteed or endorsed by the publisher.
