# Supplementary material for: Ivermectin and the Integrity of Healthcare Evidence During COVID-19
Source: Front Public Health. 2022 Mar 1;10:788972. doi: 10.3389/fpubh.2022.788972 (PMC8921859; doi:10.3389/fpubh.2022.788972)
Supplement: Supplementary file 1 [file Table_1.docx]

# Supplementary Material A: References Identified in the PubMed Search, 31 August 2021

### Systematic reviews and meta-analyses of ivermectin

Bartoszko JJ, Siemieniuk RAC, Kum E, Qasim A, Zeraatkar D, Ge L, et al. Prophylaxis against covid-19: living systematic review and network meta-analysis. BMJ (2021) 373:n949. doi: 10.1136/bmj.n949

Bhowmick S, Dang A, Vallish BN, Dang S. Safety and efficacy of ivermectin and doxycycline monotherapy and in combination in the treatment of COVID-19: a scoping review. Drug Saf (2021) 44:635-44. doi: 10.1007/s40264-021-01066-y

Bryant A, Lawrie TA, Dowswell T, Fordham EJ, Mitchell S, Hill SR, Tham TC. Ivermectin for prevention and treatment of COVID-19 infection: a systematic review, meta-analysis, and trial sequential analysis to inform clinical cuidelines. Am J Ther (2021) 28(4):e434-e460. doi: 10.1097/MJT.0000000000001402

Hill A, Garratt A, Levi J, Falconer J, Ellis L, McCann K, et al. Meta-analysis of randomized trials of ivermectin to treat SARS-CoV-2 infection. Open Forum Infectious Diseases (2021) 8:ofab358. doi: 10.1093/ofid/ofab358

Kory P, Meduri GU, Varon J, Iglesias J, Marik PE. Review of the emerging evidence demonstrating the efficacy of ivermectin in the prophylaxis and treatment of COVID-19. American Journal of Therapeutics (2021) 28:e299-e318. doi: 10.1097/MJT.0000000000001377

Kow CS, Merchant HA, Mustafa ZU, Hasan SS. The association between the use of ivermectin and mortality in patients with COVID-19: a meta-analysis. Pharmacol Rep (2021) 1-7. doi: 10.1007/s43440-021-00245-z

Padhy BM, Mohanty RR, Das S, Meher BR. Therapeutic potential of ivermectin as add on treatment in COVID 19: a systematic review and meta-analysis. J Pharm Pharm Sci (2020) 23:462-469. doi: 10.18433/jpps31457

Popp M, Stegemann M, Metzendorf M-I, Gould S, Kranke P, Meybohm P, et al. Ivermectin for preventing and treating COVID‐19. Cochrane Database of Systematic Reviews (2021) 7: CD015017. doi: 10.1002/14651858.CD015017.pub2

Roman YM, Burela PA, Pasupuleti V, Piscoya A, Vidal JE, Hernandez AV. Ivermectin for the treatment of COVID-19: a systematic review and meta-analysis of randomized controlled trials. Clin Infect Dis (2021) ciab591. doi: 10.1093/cid/ciab591

Zein AFMZ, Sulistiyana CS, Raffaelo WM, Pranata R. Ivermectin and mortality in patients with COVID-19: A systematic review, meta-analysis, and meta-regression of randomized controlled trials. Diabetes Metab Syndr (2021) 15(4):102186. doi: 10.1016/j.dsx.2021.102186

### References to articles not included for reasons summarized in the article

Banno M, Tsujimoto Y, Ishikane M. Need for more randomized controlled trials with rigorous methodology to confirm that ivermectin is not a viable option for the treatment of coronavirus disease. Clin Infect Dis (2021) ciab689. doi: 10.1093/cid/ciab689.

Heidary F, Gharebaghi R.J Ivermectin: a systematic review from antiviral effects to COVID-19 complementary regimen. Antibiot (Tokyo). 2020 Sep;73(9):593-602. doi: 10.1038/s41429-020-0336-z.

Hill A, Garratt A, Levi J, Falconer J, Ellis L, McCann K, et al. Expression of concern: “Meta-analysis of randomized trials of ivermectin to treat SARS-CoV-2 infection. Open Forum Infectious Diseases (2021) 8:ofab394. doi: 10.1093/ofid/ofab394

Kim MS, An MH, Kim WJ, Hwang TH. Comparative efficacy and safety of pharmacological interventions for the treatment of COVID-19: A systematic review and network meta-analysis. PLoS Med (2020) 17(12):e1003501. doi: 10.1371/journal.pmed.1003501

Kinobe RT, Owens L. A systematic review of experimental evidence for antiviral effects of ivermectin and an in silico analysis of ivermectin's possible mode of action against SARS-CoV-2. Fundam Clin Pharmacol (2021) 35(2):260-76. doi: 10.1111/fcp.12644

Malin JJ, Spinner CD, Janssens U, Welte T, Weber-Carstens S, Schälte G, et al. Key summary of German national treatment guidance for hospitalized COVID-19 patients. Key pharmacologic recommendations from a national German living guideline using an Evidence to Decision Framework (last updated 17.05.2021). Infection (2021) 6:1-14. doi: 10.1007/s15010-021-01645-2

O Murchu E, Spillane S, Byrne P, O’Neill M, Harrington P, Ryan M. Interventions in an ambulatory setting to prevent progression to severe disease in patients with COVID-19: a systematic review. Ann Pharmacother (2021) 10600280211028242. doi: 10.1177/10600280211028242

Rakedzon S, Neuberger A, Domb AJ, Petersiel N, Schwartz E. From hydroxychloroquine to ivermectin: what are the anti-viral properties of anti-parasitic drugs to combat SARS-CoV-2? J Travel Med (2021) 28:taab005. doi: 10.1093/jtm/taab005

Rochwerg B, Agarwal A, Siemieniuk RA, Agoritsas T, Lamontagne F, Askie L, et al. A living WHO guideline on drugs for covid-19. BMJ (2020) 370:m3379. doi: 10.1136/bmj.m3379

Rochwerg B, Kawano-Dourado L, Qadir N. How trustworthy guidelines can impact outcomes. Curr Opin Crit Care (2021) 27:544-50. doi: 10.1097/MCC.0000000000000858.

Siemieniuk RA, Bartoszko JJ, Ge L, Zeraatkar D, Izcovich A, Kum E, et al. Drug treatments for covid-19: living systematic review and network meta-analysis. BMJ (2020) 370:m2980. doi: 10.1136/bmj.m2980
